# The Role of Depression in Complications, Functional Recovery, and Healthcare Utilization Following Shoulder Arthroplasty: A Systematic Review of Contemporary Literature

**DOI:** 10.7759/cureus.92465

**Published:** 2025-09-16

**Authors:** Iosafat Pinto, Konstantinos Ditsios, George Paraskevas, Chrysanthos Chrysanthou

**Affiliations:** 1 Department of Orthopaedics and Trauma, General Hospital of Imathia, Health Unit of Veria, Veria, GRC; 2 2nd Orthopaedic Department of Aristotle University of Thessaloniki, Aristotle University of Thessaloniki, Thessaloniki, GRC; 3 Department of Anatomy and Surgical Anatomy, Faculty of Health Sciences, Medical School, Aristotle University of Thessaloniki, Thessaloniki, GRC; 4 Department of Occupational Therapy, University of Western Macedonia, Kozani, GRC

**Keywords:** complications, depression, functional outcomes, healthcare utilization, mental health, shoulder arthroplasty

## Abstract

Depression is increasingly recognized as an important factor influencing outcomes after total shoulder arthroplasty (TSA) and reverse shoulder arthroplasty (RSA), yet its specific impact remains incompletely defined. This systematic review, conducted in accordance with Preferred Reporting Items for Systematic Reviews and Meta-Analyses (PRISMA) guidelines, synthesized evidence from 14 original studies published in the last decade examining the association between depression and postoperative outcomes following TSA and RSA. Across diverse institutional and national datasets, depressed patients generally demonstrated lower absolute patient-reported outcome scores, though several studies reported comparable relative improvements from baseline. Depression was consistently associated with higher complication rates, including infection, thromboembolic events, and mechanical failure, as well as prolonged opioid use, greater emergency department utilization, increased readmissions, longer length of stay, and higher healthcare costs. Most studies were of moderate to high methodological quality on the Newcastle-Ottawa Scale, with no high-risk studies identified. The findings suggest that depression independently contributes to worse objective and patient-reported outcomes after shoulder arthroplasty, but does not preclude meaningful functional improvement. Routine preoperative depression screening and mental health optimization may represent modifiable targets to enhance surgical recovery, reduce complications, and lower healthcare utilization in this population.

## Introduction and background

Total shoulder arthroplasty (TSA) and reverse shoulder arthroplasty (RSA) are well-established procedures for managing glenohumeral osteoarthritis, complex fractures, and rotator cuff arthropathy [1,2]. As these procedures become more prevalent and patient expectations for pain relief and functional improvement continue to rise [3], attention has shifted to identifying modifiable predictors of postoperative outcomes. Among these, the role of psychological health, particularly depression, has emerged as an area of growing clinical importance.

Depression is a widely recognized psychiatric disorder that affects over 300 million individuals worldwide and is defined by the World Health Organization as a common mental disorder characterized by persistent sadness, loss of interest, and associated emotional and physical symptoms, with its diagnosis becoming increasingly common among patients preparing for elective surgery [4]. In the context of total joint arthroplasty, particularly of the hip and knee, depression has been associated with worse postoperative outcomes, including higher complication rates, increased healthcare utilization, and inferior patient-reported outcomes [5,6]. However, its influence on the outcomes of shoulder arthroplasty is less well-characterized, despite the distinct biomechanical demands and rehabilitative trajectories associated with TSA and RSA.

Emerging evidence suggests that patients with depression may experience more postoperative pain, require greater opioid use, and have higher rates of complications and readmission following shoulder arthroplasty [7,8]. The interpretation and clinical application of these findings are complicated by substantial heterogeneity in study design, sample size, and the operationalization of depression. Furthermore, it remains uncertain whether depression independently contributes to adverse outcomes or merely reflects underlying health and sociodemographic vulnerabilities.

Given the clinical and health system implications, a comprehensive understanding of how depression influences outcomes following TSA and RSA is urgently needed. This systematic review was conducted to synthesize the current evidence on the association between depression and postoperative functional outcomes, complications, healthcare utilization, and the extent to which depression is an independent risk factor in shoulder arthroplasty populations. Furthermore, it examines whether identifying and optimizing mental health preoperatively may offer a pathway to improving surgical outcomes.

## Review

Methods

This systematic review was conducted in accordance with the Preferred Reporting Items for Systematic Reviews and Meta-Analyses (PRISMA) guidelines and was prospectively registered on the Open Science Framework (OSF) (Registration DOI: https://doi.org/10.17605/OSF.IO/T65XA). The primary objective was to evaluate the impact of depression on postoperative outcomes following TSA and RSA.

A comprehensive search of PubMed, CINAHL, and Cochrane Libraries was performed, limited to studies published in the last 10 years. The search strategy employed a Boolean query combining terms related to the procedure, outcomes, complications, and depression: (arthroplasty OR replacement) AND shoulder AND (outcome OR complication) AND depression. The search was last updated on August 1, 2025. Only peer-reviewed, full-text original studies published in English were considered.

Eligibility criteria were structured using the Population, Intervention, Comparison, Outcome (PICO) framework. The population included adult patients undergoing primary TSA or RSA. The intervention of interest was the presence of a diagnosed depressive disorder or a documented history of depression, either self-reported, clinically diagnosed, or defined via coding systems. Studies were included if they evaluated depression either as a primary exposure or as a variable in multivariate analyses related to postoperative outcomes. While most studies included a defined comparison group without depression, others analyzed depression as an independent predictor within a single cohort. The outcomes of interest included postoperative functional results, complication rates, opioid use, and healthcare utilization.

Studies were included if they (1) involved patients undergoing primary TSA or RSA, (2) evaluated depression as an exposure variable or included it as an independent predictor in outcome analyses, and (3) reported outcomes postoperatively. Studies were excluded if they were reviews, editorials, case reports, technical notes, or if the full text was unavailable. Additionally, studies were excluded if they failed to distinguish outcomes based on depressive status.

Following the database search, two reviewers (I.P. and C.C.) independently screened all resulting titles and abstracts for relevance. Full-text reviews were performed on all potentially eligible articles. The references of all included studies and review articles were also manually cross-referenced to verify that no relevant articles were missing. Any discrepancies in data extraction were resolved through discussion, and if appropriate, the senior author was consulted (K.D.). Data extraction was performed independently by two reviewers (I.P. and C.C.) and included study design, sample size, depression definition, outcome measures, complications, healthcare utilization, and key findings. Interrater agreement for study inclusion was measured using Cohen’s kappa, with a k score of over 0.81 indicating almost perfect agreement, 0.61-0.80, substantial agreement; 0.41-0.60, moderate agreement; 0.21-0.40, fair agreement; and 0.20 or less, slight agreement [9]. Due to heterogeneity in study designs, outcome reporting, and definitions of depression, a meta-analysis was not feasible. Instead, a qualitative synthesis of the evidence was performed, with outcomes grouped by domain. No statistical conversions were performed. All extracted data were presented as reported by the study authors. Missing summary statistics were not imputed. Results of individual studies were tabulated according to reported outcome domains such as functional scores, complications, opioid use, and healthcare utilization. Summary tables were created to present study characteristics, outcome measures, and key findings. The study selection process was also displayed in a flow diagram.

To assess the methodological quality of the included studies, we used the Newcastle-Ottawa Scale (NOS), a validated tool for evaluating non-randomized studies [10]. Each study was independently assessed across three domains: selection of study groups (maximum 4 stars), comparability of cohorts (maximum 2 stars), and ascertainment of the outcome (maximum 3 stars), yielding a total score out of 9. Studies scoring ≥7 were considered to have low risk of bias, scores between 5 and 6 indicated moderate risk, and scores <5 were considered high risk. This process was conducted to provide transparency regarding the quality and internal validity of the included literature.

Results

**Figure 1:**
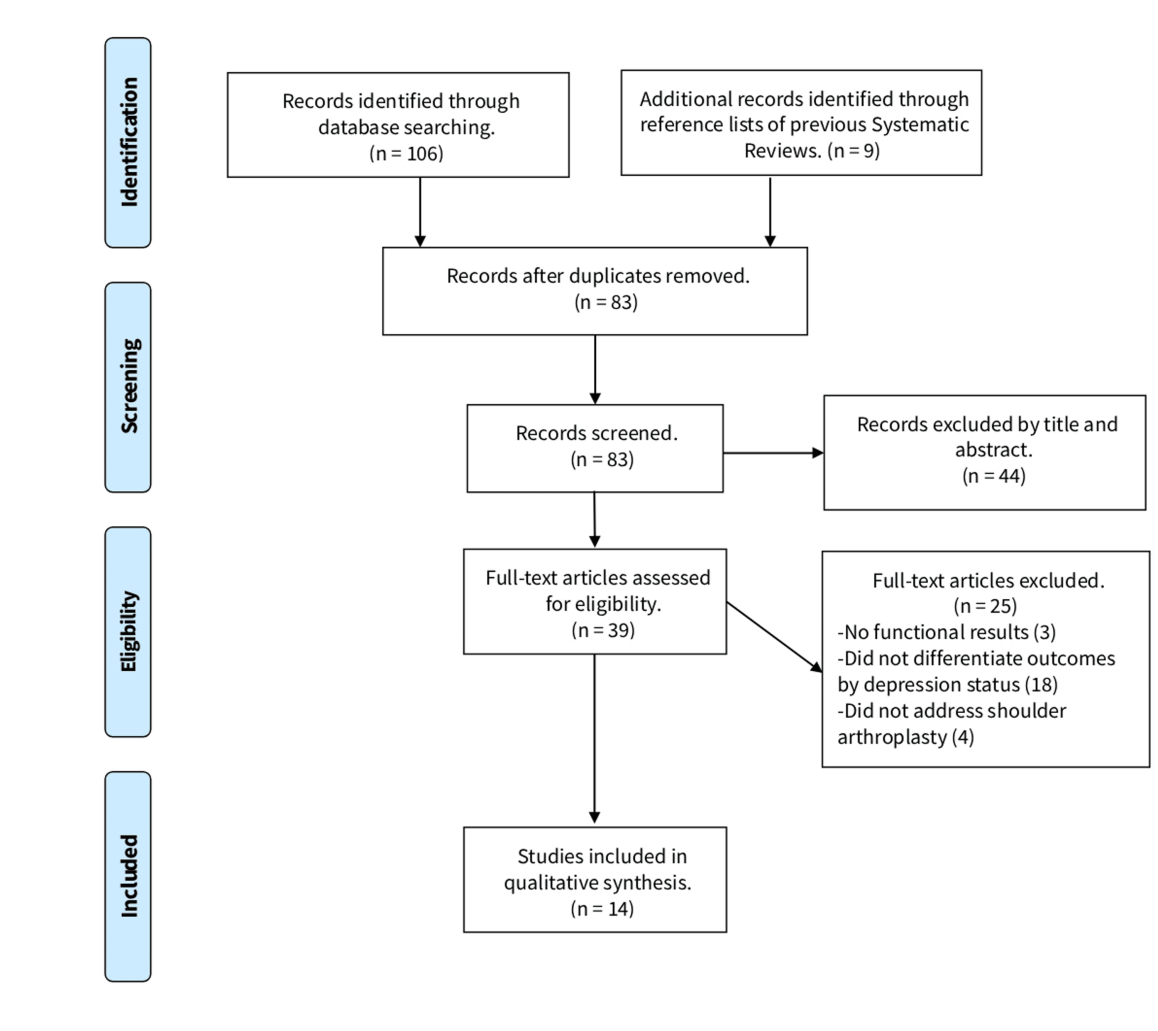
PRISMA flow diagram depicting the flow of information through the different phases of research PRISMA: Preferred Reporting Items for Systematic Reviews and Meta-Analyses

A total of 14 studies met the inclusion criteria for this systematic review [7,8,11-22], and the process of selection is summarized in a PRISMA flow diagram (Figure 1). There was substantial agreement between reviewers during the title and abstract screening stage (k = 0.746), and almost perfect agreement during full-text review (k = 0.839). Sample sizes varied considerably across the included studies, reflecting both institutional and national-level datasets. Depression was commonly identified through administrative diagnostic codes or antidepressant medication use. Among studies that included both depressed and non-depressed cohorts, the reported prevalence of depression ranged from 9% to 50%, and the proportion of female patients in these cohorts was generally higher, often exceeding 60%. The characteristics of the included studies are summarized in Table 1.

**Table 1:** Characteristics of studies included in the systematic review Abbreviations: TSA = Total Shoulder Arthroplasty; RSA = Reverse Shoulder Arthroplasty; ICD = International Classification of Diseases; PHQ-9 = Patient Health Questionnaire-9; M = Males; F = Females

Author (Year)	Study Design	Level of Evidence	Sample Size	Depression Definition	Mean Age	Gender (M/F)	Implant Type
Werner et al. (2016) [12]	Retrospective case series	IV	150	Medical record	71.6	48/102	RSA
Werner et al. (2017) [7]	Retrospective cohort	III	264	ICD codes	67.2	93/171	TSA
Diamond et al. (2023) [8]	Retrospective database analysis	III	24326	ICD codes	67.5	11516/12804	RSA
Wong et al. (2018) [17]	Retrospective design	II	280	Medical record	66.7	142/138	TSA, RSA
Lunati et al. (2020) [20]	Retrospective database analysis	III	22623	ICD codes	69.4	11145/11478	TSA,
MacFarlane et al. (2024) [13]	Prospective cohort	II	93	PHQ-9	69.1	45/48	TSA, RSA
Mayfield et al. (2024) [15]	Retrospective database analysis	III	49997	ICD codes	66.2	31,998/17,999	TSA, RSA
Liu et al. (2025) [11]	Retrospective cohort	III	1169	ICD codes	68	513/656	TSA, RSA
Gordon et al. (2025) [18]	Retrospective database analysis	III	21335	Screening test	67.3	6725/ 14610	TSA, RSA
Mollon et al. (2016) [14]	Retrospective database analysis	III	224060	ICD codes	65.2	123,905/100155	TSA
Lu et al. (2023) [19]	Retrospective database analysis	III	34198	ICD codes	68.7	16234/17964	TSA
Khela et al. (2025) [16]	Retrospective database analysis	III	24743	ICD codes	65.2	11586/3157	TSA
Colasanti et al. (2023) [21]	Retrospective cohort	III	596	ICD codes	63.5	239/335	TSA, RSA
Swiggett et al. (2021) [22]	Retrospective database analysis	III	113648	ICD codes	71	50446/20310	TSA

Patient-reported outcome measures (PROMs) were mostly assessed using the American Shoulder and Elbow Surgeons (ASES) score, with depressed patients commonly demonstrating lower absolute postoperative values compared to their non‑depressed counterparts. Detailed comparisons are presented in Table 2. The Colasanti et al. study found that patients with depression undergoing shoulder arthroplasty, whether TSA or RSA, had significantly lower postoperative functional scores, less improvement from baseline, lower satisfaction rates, and higher adverse event rates compared to patients without a psychiatric diagnosis [21]. These negative associations were present regardless of whether patients were taking psychiatric medication. Similarly, Werner et al. demonstrated that depression independently predicted both lower final ASES scores and smaller improvements in ASES scores following total shoulder arthroplasty [7]. Of note, MacFarlane et al. showed a statistically significant decline in postoperative pain control and greater dissatisfaction among patients with higher mental health burden [13].

**Table 2:** Functional outcomes in patients with depression Abbreviations: ASES = American Shoulder and Elbow Surgeons score; ROM = Range of Motion; VAS = Visual Analog Scale; SF-12 = 12-Item Short Form Survey; PHQ-SADS = Patient Health Questionnaire–Somatic, Anxiety, and Depressive Symptoms; PCS = Pain Catastrophizing Scale; QuickDASH = Quick Disabilities of the Arm, Shoulder, and Hand; PROMIS-10 = Patient-Reported Outcomes Measurement Information System–10; CS = Constant score; SASS = Shoulder Arthroplasty Smart Score; PROMIS-MH = Patient-Reported Outcomes Measurement Information System Mental Health

Study	Functional Outcome	Finding in Depressed Patient	Statistical Significance (p value)
Wong et al. (2018) [17]	ASES, SF-12, VAS, ROM	Similar improvements but lower absolute scores	0.6, 0.28, 0.8, 0.3
MacFarlane et al. (2024) [13]	PHQ-SADS, PCS	Greater pain and dissatisfaction	0.002
Werner et al. (2016) [12]	ASES	Poor improvement	0.005
Werner et al. (2017) [7]	ASES, SF-12	Less improvement	0.001, 0.006
Liu et al. (2025) [11]	ASES, QuickDASH, PROMIS-10	No significant difference	0.15
Colasanti et al. (2023) [21]	ASES, PROMIS-MH, CS, SASS	Lower postoperative outcome scores	0.0001

Opioid utilization was significantly higher among patients with depression. Mayfield et al. found that depression was associated with prolonged opioid use beyond the perioperative window, and this association persisted even after controlling for surgical complexity and baseline pain scores [15]. Furthermore, MacFarlane et al. confirmed that patients with greater psychological burden consumed more opioids postoperatively and were more likely to require refills, further emphasizing the complex interplay between mental health and pain management [13].

Complication rates were significantly elevated in depressed patients across multiple studies, and these findings are summarized in Table 3. Lunati et al. demonstrated that the following complications were more common in patients with depression: sepsis, revision within one year, prosthetic joint infection within one year, return to the operating room for irrigation and debridement, prosthetic complication, and wound complication [20]. Additionally, they reported that depression independently predicted increased 90-day readmission, with an adjusted odds ratio (OR) of 1.55 and a 95% confidence interval. In addition, Khela et al. reported higher rates of postoperative complications, including deep infection and revision surgery, among depressed patients [16]. Gordon et al. noted a marked increase in both implant complications and medical readmissions among depressed patients, although the presence of a preoperative screening program was associated with a reduced overall medical and implant complication burden [18].

**Table 3:** Complications and healthcare utilization in patients with depression Abbreviations: LOS = Length of Stay

Study	Complication Type	Findings in Depressed Patients	Statistical Significance (p value)
Lunati et al. (2020) [20]	90-day complications	Higher 90-day complication rate	<0.05
Gordon et al. (2025) [18]	Medical + implant complications	More complications if no screening program	0.001
Liu et al. (2025) [11]	90-day complications, 365-day mortality	No significant difference	0.15
Mollon et al. (2016) [14]	Medical complications	Higher rate of delirium, anemia, and infection	0.001
Mayfield et al. (2024) [15]	90-day complications, Opioid consumption	More complications, Higher opioid use	0.001
MacFarlane et al. (2024) [13]	Pain, dissatisfaction, opioid refills	Greater dissatisfaction, higher opioid utilization	0.002
Lu et al. (2023) [19]	In-hospital prosthesis-related complications	Elevated prosthetic complication rates	0.03
Khela et al. (2025) [16]	Implant-related complications	Higher irrigation and debridement rates	0.001
Diamond et al. (2023) [8]	LOS, 90-day medical complications, 90-day episode of care costs	Longer LOS, higher rates of complications, increased costs of care	0.001
Swiggett et al. (2021) [22]	Complications and readmission rates	Longer LOS, higher rate of readmissions, medical and implant-related complications	0.0001
Colasanti et al. (2023) [21]	Adverse events	Infections, glenoid dissociation, and glenoid aseptic loosening	0.05

Healthcare utilization outcomes, including readmissions, emergency department visits, and length of stay, were consistently worse in the depression group. Diamond et al. reported that patients with depressive disorder demonstrated substantially higher healthcare utilization following primary reverse shoulder arthroplasty [8]. Specifically, those with depression were more likely to present to the emergency department (ED) within 90 days postoperatively, and this effect was magnified among patients who had preoperative ED visits. Additionally, the depressive disorder cohort incurred higher mean 90‑day episode-of-care costs compared to non-depressed patients, reflecting increased resource utilization in the early postoperative period. Swiggett et al. reported that patients with depressive disorders undergoing primary TSA had longer hospital stays, higher 90‑day readmission rates, and greater odds of medical complications, including anemia, cerebrovascular events, acute kidney failure, and pneumonia [22]. Over two years, they also experienced more implant-related complications, such as periprosthetic fractures, dislocation, and infection, as compared to non‑depressed controls.

Only two studies did not demonstrate statistically significant differences in outcomes and complications by depression status. The Liu et al. study, which involved a relatively small sample size and limited follow-up, could not identify any association between depression and shoulder arthroplasty regarding 90-day reoperation of the same joint, 365-day mortality, or patient-reported outcomes [11]. Also, the Wong et al. study, which included a mixed cohort of patients with various mental health conditions rather than depression alone, claimed similar improvements in pain, function, and range of motion after shoulder arthroplasty in patients with and without diagnosed mental health conditions [17]. 

Of the 14 included studies, 12 were rated as a low risk of bias (scores ≥7), and two were rated as a moderate risk (scores 5-6). No studies met the criteria for high risk. Most studies demonstrated robust participant selection and outcome ascertainment. Similarly, exclusion of studies with mixed mental health cohorts (where depression was not analyzed separately) did not materially alter the overall direction of associations reported across the literature. However, several studies lacked sufficient adjustment for potential confounders, such as anxiety, substance use, or chronic pain, limiting comparability between cohorts. While no clear evidence of selective reporting was identified, variations in the scope of reported outcomes mean that the risk of bias due to missing results cannot be completely excluded. A detailed summary of NOS scoring is provided in Table 4.

**Table 4:** Risk of bias assessment using the Newcastle–Ottawa scale

Study	Selection (max 4)	Comparability (max 2)	Outcome (max 3)	Total Score (out of 9)	Risk of Bias
Werner et al. (2017) [7]	4	2	3	9	Low
Diamond et al. (2023) [8]	4	3	2	9	Low
Wong et al. (2018) [17]	3	2	3	8	Low
Lunati et al. (2020) [20]	4	2	3	9	Low
MacFarlane et al. (2024) [13]	4	2	3	9	Low
Mayfield et al. (2024) [15]	3	2	3	8	Low
Liu et al. (2025) [11]	4	2	3	9	Low
Gordon et al. (2025) [18]	4	2	2	8	Low
Mollon et al. (2016) [14]	3	1	2	6	Moderate
Lu et al. (2023) [19]	4	2	3	9	Low
Khela et al. (2025) [16]	3	2	3	8	Low
Werner et al. (2016) [12]	2	1	2	5	Moderate
Colasanti et al. (2023) [21]	4	2	3	9	Low
Swiggett et al. (2021) [22]	4	2	2	8	Low

Discussion

This systematic review demonstrates a consistent and clinically meaningful association between depression and adverse outcomes following total and reverse shoulder arthroplasty. Across 14 original studies published within the last decade and involving over 500,000 patients, depression was linked to higher complication rates, increased opioid use, worse patient-reported outcomes, and greater healthcare utilization. These findings align with broader surgical literature, where psychological comorbidities have long been recognized as important predictors of postoperative recovery [23,24].

Our decision to restrict inclusion to studies from the last 10 years was guided by the rapid evolution of shoulder arthroplasty techniques and implants during this time. Advances in prosthetic design, surgical navigation, and perioperative management have improved both the consistency and quality of outcomes [25]. Reverse shoulder arthroplasty, for instance, has become more biomechanically efficient and durable, making comparisons with older techniques less clinically meaningful. By focusing on recent studies, we aimed to provide a synthesis that reflects current surgical standards and avoids data skewed by outdated practices.

An important nuance emerging from the included studies is that although patients with depression often present lower absolute postoperative functional scores, as compared to non‑depressed individuals, this difference does not always represent a clinical importance [7]. Evidence indicates that while the absolute values may be lower, the relative improvement from baseline is frequently similar between groups, indicating that patients with depression can still derive substantial functional benefit from surgery [17]. This suggests that a diagnosis of depression alone should not discourage patients from undergoing shoulder arthroplasty when otherwise indicated, as several studies demonstrated comparable relative improvements from baseline despite lower absolute scores. These patients can achieve substantial functional benefit, provided that expectations are appropriately managed. This underscores the importance of preoperative counseling and mental health optimization, not only to support recovery but also to help patients engage more effectively with rehabilitation and pain management strategies.

Despite the potential for meaningful improvement, depression was also associated with higher rates of wound complications, infections, thromboembolic events, and even mechanical failure in some studies [18,20]. Such associations are supported by biologically plausible mechanisms, including neuroinflammatory pathways, immune dysregulation, and behavioral factors that may impair healing [26,27]. Moreover, opioid consumption was also notably higher among patients with depression [13,15], a finding of particular concern given ongoing efforts to reduce opioid prescribing in orthopedic surgery. Depression may lower pain thresholds and amplify nociceptive signaling, thereby increasing analgesic requirements and the risk of long-term dependence [28]. Behavioral factors, such as self-medication to cope with pain and mood symptoms, may further contribute to this pattern.

Greater healthcare utilization further reflects the impact of depression in this review. Lu et al. reported depression as an independent risk factor for prosthesis-related complications, underscoring its relevance to adverse outcomes and resource use in the TSA population, whereas Gordon et al. found that depression predicted readmission and implant complications, particularly in those without formal mental health screening [18,19]. Importantly, the presence of structured screening and optimization pathways was associated with lower complication rates and reduced costs, supporting the feasibility and benefit of integrating mental health services into preoperative care.

These findings align with prior reviews, which also reported worse outcomes in patients with mental health conditions undergoing shoulder arthroplasty [29-32]. Unlike these earlier syntheses, which often included older studies or combined data from different joints, our review focuses exclusively on contemporary shoulder arthroplasty literature.

Our review has a number of limitations inherent to the included studies. Definitions of depression varied, ranging from diagnostic codes and medication history to validated screening tools. Comorbid mental health conditions, such as anxiety or substance use disorders, may have confounded results but were not separated in subgroup analyses. This review has also some process‑related limitations. Although a comprehensive search strategy was applied across multiple databases, it is possible that relevant studies indexed in other databases or unpublished data were missed. Moreover, restricting inclusion to studies published in English may have introduced a language bias. In spite of those limitations, our study also has several strengths. To our knowledge, it is the first synthesis focusing exclusively on original studies from the past decade, thereby reflecting modern implant designs and surgical techniques. Also, the consistency of observations across diverse settings, study designs, and patient populations strengthens the conclusion that depression has a distinct and measurable impact on shoulder arthroplasty outcomes. It should also be noted that most included studies were of moderate to high methodological quality, supporting the robustness of our review. Finally, no substantial amendments were made to the objectives, eligibility criteria, or planned methods after registration in OSF.

## Conclusions

This systematic review highlights depression as a prevalent and clinically significant comorbidity that adversely affects outcomes following total and reverse shoulder arthroplasty. Across the included studies, depression was consistently associated with higher complication rates, greater opioid consumption, lower patient‑reported outcome scores, and increased healthcare utilization. While absolute functional scores were often lower in patients with depression, several studies demonstrated comparable relative improvements from baseline, indicating that these patients can still achieve meaningful benefits from surgery.

These findings underscore the importance of recognizing and addressing depression in the perioperative period. Routine screening and targeted optimization of mental health may not only improve patient satisfaction and functional recovery but also reduce complications, readmissions, and healthcare costs. In clinical practice, this could involve incorporating validated screening tools into preoperative pathways, with appropriate referral or intervention when indicated. Future research should focus on prospective studies to clarify causal mechanisms and to evaluate whether preoperative mental health interventions can modify these risks and enhance surgical outcomes in this vulnerable population.
